# Community treatment for adolescents with mental health problems

**DOI:** 10.1192/j.eurpsy.2021.1699

**Published:** 2021-08-13

**Authors:** A. Diaz-Caneja, A. Hernández, M.I. Toral

**Affiliations:** 1 Day Hospital For Children And Adolescents, Servicio Murciano Salud, Cartagena, Spain; 2 Day Hospital For Children And Adolescents, Servicio Murciano salud, Cartagena, Spain

**Keywords:** Community treatment, adolescents, mental health

## Abstract

**Introduction:**

This is the case of a 15-year-old boy who had been socially isolated in his house for over 1 year. He had become increasingly agitated, but refused any help offered.

**Objectives:**

To establish the role of community treatment in adolescents with mental health problems.

**Methods:**

Summary of the interventions taken place during the treatment

**Results:**

Initially this young person refused any medical treatment, so we tried first supportive therapy and CBT. He dicho not obtain any benefits as he appeared experiencias paranoid ideation and thoughts of being persecuted in the streets. With support from the occupational therapist, the young person started to take care of his personal hygiene. Afterwards he started to take oral medication with partial response. We decided to switch to im treatment. In conjunction with CBT, the young person was gradually able to llaves the house. All the sessions during the first few months took place at his family home. These visits were weekly or twice weekly. Once he left the house, he attended the grupos at the day hospital. After 18 months, he was discharged without medication and he is currently studying for a degree.

**Conclusions:**

Community treatment in adolescent with mental health problems is a better opción to establish good rapport and avoid stressful situations that could take place in an in-patient facilita.

**Disclosure:**

No significant relationships.

